# Barriers and Enablers to Pre‐Registration Nurses Providing Safe Care for Individuals Experiencing Suicidal Distress: A Scoping Review

**DOI:** 10.1111/jan.70274

**Published:** 2025-09-30

**Authors:** Renee Molloy, Brent Hayward, Samantha Scott, Alison Hansen, Adam Searby

**Affiliations:** ^1^ Monash University School of Nursing and Midwifery Melbourne Victoria Australia

**Keywords:** nursing assessment, nursing students, suicidal ideation, suicide, suicide prevention

## Abstract

**Aims:**

To identify research gaps by mapping what is known about the barriers and enablers to pre‐registration nursing students identifying signs of suicidal distress in healthcare consumers and providing clear pathways of support.

**Design:**

Scoping review.

**Methods:**

This scoping review was conducted using Arksey and O'Malley's (2005) five stage framework and the Levec et al. (2010) extensions of this framework.

**Data Sources:**

The Cumulative Index of Nursing and Allied Health Literature (CINAHL) Complete and Ovid MEDLINE databases were searched to identify relevant articles, keywords and search terms to inform the full search strategy for CINAHL. This search strategy was then adapted for Scopus, PsychInfo, Emcare, Medline and ERIC, searched in November 2024.

**Results:**

Studies eligible for inclusion (*N* = 28) represented research from 14 countries; most (53.5%, *n* = 15) used a quantitative design, 11 (39.3%) were qualitative and two (7.1%) used a mixed‐methods design. Barriers found from the scoping review included a low level of knowledge of suicidality, stigma preventing students from assessing and acting on suicidal ideation, and a lack of confidence in providing care to healthcare consumers expressing suicidality. Enablers included lived experience, exposure to individuals expressing suicidal ideation and education, simulation and role play. This review also contributes to the existing literature about the relationship of nursing to existing suicide prevention frameworks and suggests revision of these frameworks to address staff attitudes and beliefs, as well as lived and living experience.

**Conclusion:**

Nurses are ideally placed to assess and respond to suicidality among healthcare consumers, and preparation should begin during pre‐registration studies. Our scoping review indicates that further research work is needed to address the barriers to working with healthcare consumers expressing suicidality and to enhance the enablers to provide safe care.

**Implications for the Profession and/or Patient Care:**

Addressing the barriers and enablers to pre‐registration nursing students providing safe care for healthcare consumers expressing suicidality is essential. Further research is required to address the barriers and enhance the enablers identified in this scoping review.

**Impact:**

- What problem did the study address? This scoping review summarised the literature on pre‐registration student ability to work with healthcare consumers expressing suicidality, identifying barriers and enablers.
- What were the main findings? Barriers include poor knowledge of suicidality, stigma, fear and a lack of confidence in working with healthcare consumers expressing suicidality. Enablers include lived experience, exposure to clinical settings where healthcare consumers express suicidality and simulation and education.
- Where and on whom will the research have an impact? The research will have an impact on providers of pre‐registration nursing degrees, where the inclusion of content addressing suicidality and exposure to settings where individuals express suicidal ideation is shown to improve attitudes and knowledge of suicidality assessment.

**Reporting Method:**

PRISMA checklist for scoping reviews.

**Patient or Public Involvement:**

This study did not include patient or public involvement in its design, conduct or reporting.

**Summary**

- What does this paper contribute to the wider global clinical community?
  ○ This paper provides insights on barriers and enablers to pre‐registration nursing students assessing and working with healthcare consumers who express suicidality, in addition to providing a foundation for future work to address these barriers and enhance enablers.

## 1 Introduction

Despite being largely preventable, suicide is a major health concern throughout the world (Pirkis et al. 2024). Globally, more than 700,000 people died by suicide in 2019, with 58% of suicides happening before the age of 50 years, and suicide being the fourth leading cause of death among persons aged 15–29 years (World Health Organisation 2021a). The World Health Organisation (2021b) specifies nurses as one professional group who should be recipients of capacity‐building for suicide prevention. While research has conceptualised how to support nurses to respond to suicide and suicidal patients (Talseth and Gilje 2011), there has been no synthesis of literature about how to prepare and support pre‐registration nurses to do this. Pre‐registration nursing students, representing the next wave of nurses, are an ideal target group to build capacity in responding to people with suicidal distress given their numbers and presence in all healthcare settings.

## 2 Zero Suicide Framework

As suicide remains a key mental health priority for both local and federal governments, many health services have looked to the Zero Suicide Framework (ZSF) as a way of supporting staff, including nurses, to respond to people with suicidal distress. The ZSF uses the seven essential elements of suicide care to guide healthcare system transformation: (1) Lead, (2) Train, (3) Identify, (4) Engage, (5) Treat, (6) Transition and (7) Improve (Zero Suicide Framework 2024a). It has been implemented in several health systems (see Roth et al. 2024) despite a lack of robust evidence for its effectiveness (Mokkenstorm et al. 2018). This can be partly explained by the challenges of real‐world implementation of the framework (e.g., Boudreaux et al. 2022).

## 3 Nurses and the Zero Suicide Framework

As nurses are the largest health workforce globally (Boniol et al. 2022), the role of nurses in the ZSF is essential to implementing it effectively, yet few studies have explored this. Elevating the role of nurses in the framework over the empowerment of people with suicidal distress has been questioned (Porter et al. 2022), and implications of the framework for nurses have only been explored in the context of emergency departments (Larkin et al. 2024; Roth et al. 2024). Training informs the identification, engagement and treatment elements of the ZSF, and is key to building capacity in suicide prevention (World Health Organisation 2021b). Therefore, it is essential that the training and education needs of cohorts of the health workforce are specifically addressed, with the confidence and practice of pre‐registration nurses in responding to healthcare consumers at risk of suicide reported to be responsive to education (e.g., Vedana et al. 2018). The ZSF provides a conceptual structure for such an undertaking, and using the ZSF in this way also contributes to further knowledge about the ZSF and its relationship to nursing. However, there are limitations to applying the ZSF to pre‐registration nurses, such as the elements of ‘treat’ and ‘transition’, which refer to the implementation of evidence‐based treatment of suicidal thoughts and behaviours, and moving individuals through care with ‘warm’ handovers and supportive contacts respectively (Safer Care Victoria 2024). Despite these limitations, the ZSF outlines treating and transitioning through care as vital skills to improve responses to individuals with suicidal thoughts and behaviours, and as discussed in the next section, areas that pre‐registration nurses should be prepared in to respond.

### 3.1 Preparing Pre‐Registration Nurses

A recent systematic review by Ferguson et al. (2020) found that although there is some evidence to suggest that suicide prevention education programs do lead to increased skills, abilities, self‐confidence and attitudes among nursing students, the impact of this strategy is still not fully understood, and additional research is needed into the barriers and enablers to translating their suicide prevention education into practice. Several studies have identified the challenges to pre‐registration nurses assessing and responding to suicidal behaviour; however, to date these findings have not been summarised. For example, de Albuquerque et al. (2022) identified that nursing students felt helplessness when faced with suicidal behaviour and were reluctant to assess due to a perception that questioning would induce suicide. Scheckel and Nelson (2014) found that some students considered suicide a taboo topic, making it difficult for them to discuss. Conversely, Vedana et al. (2020) found that students with a lived experience of suicide have less moralistic attitudes towards people with suicidal behaviour.

To ensure that suicide intervention is tailored to the needs of pre‐registration nurses, an exploration of the barriers and enablers to pre‐registration nursing students identifying individuals with suicidal distress, engaging with individuals at risk of suicide, and treating suicidal thoughts and behaviours (the identify, engage and treat elements of the ZSF) is essential. A scoping review is a suitable way to identify these barriers and enablers, and the gaps in this knowledge (Munn et al. 2018). The results can also be used to inform the ongoing development of the ZSF and its application to nursing practice.

## 4 The Review

### 4.1 Aim

The aim of this scoping review is to identify research gaps by mapping what is known about barriers and enablers to pre‐registration nursing students identifying signs of suicidal distress in healthcare consumers and providing clear pathways of support. The secondary aim is to identify how these results can inform the development of the ZSF for pre‐registration nursing students.

## 5 Methods

### 5.1 Design

This scoping review was conducted using Arksey and O'Malley's (2005) five‐stage framework and the Levac et al. (2010) extensions of this framework. Scoping reviews are typically used to map or to explore the ‘conceptual boundaries’ of a research topic (Peters et al. 2024), and given our aims of mapping the barriers and enablers to pre‐registration students identifying signs of suicidal distress, and the diversity of methodological approaches found during preliminary searches, we felt the scoping review was more appropriate than a systematic approach. The five‐stage framework includes the following stages: (1) Identifying the research question (2) Identifying relevant studies (3) Study selection (4) Charting the data (5) Collating, summarising and reporting results. In addition, the PRISMA Extension for Scoping Reviews (PRISMA‐ScR) statement has been used (Tricco et al. 2018) to guide the reporting of this review. The protocol for this review has been registered on the Open Science Framework (https://osf.io/px4nr).

### 5.2 Identifying the Research Question

The *population, concept, context* (PCC) mnemonic (Peters et al. 2024) was used to guide the research question. The population included students currently enrolled in pre‐registration nursing courses. This included both comprehensive and speciality courses (e.g., mental health nursing) and students studying at all levels of the course. The *concept* to be examined was barriers and enablers to (1) identifying suicide risk, (2) engaging with individuals at risk of suicide, (3) engaging in and offering treatment. These are core elements of the ZSF. The *context* included undergraduate courses in all geographical locations. The primary research question guiding this review was, what is known about the barriers and enablers to pre‐registration nursing students identifying signs of suicidal distress and providing clear pathways of support? The secondary research questions were:

1. What are the barriers and enablers to pre‐registration nursing students identifying individuals experiencing with suicidal thoughts?
2. What are the barriers and enablers to pre‐registration nursing students engaging with individuals experiencing suicidal thoughts?
3. What are the barriers and enablers to pre‐registration nursing students treating suicidal thoughts and behaviour and/or assisting with transition to care?

### 5.3 Identifying Relevant Studies

A three‐step process, in collaboration with a university librarian, was used to identify relevant studies (Peters et al. 2024). Firstly, the Cumulative Index of Nursing and Allied Health Literature (CINAHL) Complete and Ovid MEDLINE databases were searched to identify relevant articles, keywords and search terms to inform the full search strategy for CINAHL (Appendix S1). This search strategy was then adapted for Scopus, PsychInfo, Emcare, Medline and ERIC. Secondly, these databases were searched for peer‐reviewed sources. A search for grey literature was conducted in ProQuest Dissertations and Theses Global Database and using the Google search engine. Results from Google were limited to the first 200 results (Haddaway et al. 2015). Thirdly, a search of the reference list of included studies was examined for additional relevant studies. Studies were excluded if they were not published in English. There was no date range for inclusion. While the ZSF was used to structure this scoping review, we did not use the ZSF itself and an inclusion or exclusion criterion for the search, given the sparsity of its use in published literature on suicide prevention. There is a diversity of models used in the literature of suicide prevention preparation, and limiting this literature to the ZSF would mean excluding studies relevant to the aims of this review.

### 5.4 Study Selection

**FIGURE 1:**
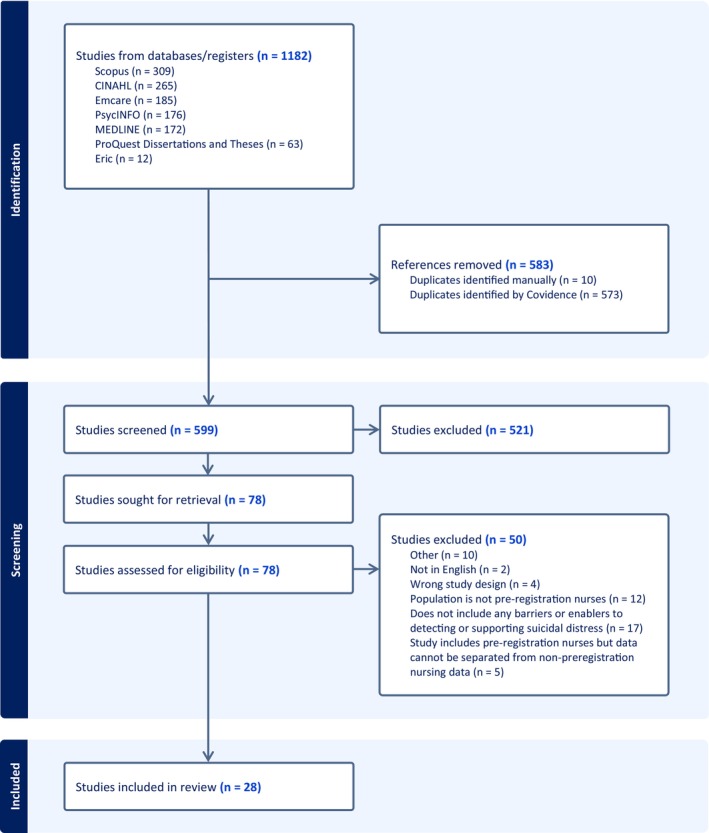
PRISMA flowchart.

All articles identified from databases were imported into Covidence (Veritas Health Innovation, Melbourne, Australia) for screening (see Figure 1). Following the removal of duplicates, the title and abstract of each article were screened by two independent reviewers against the inclusion and exclusion criteria (see Table 1). Conflicts were resolved through discussion between reviewers. Eligible articles were subject to full‐text screening by two independent reviewers (RM, SS), and conflicts were resolved through discussion with a third reviewer (AH).

**TABLE 1:** Inclusion and exclusion criteria.

Inclusion criteria	Exclusion criteria
Study exploring barriers or enablers to supporting suicidal distress or providing clear pathways of support.	Study does not include any barrier or enablers to supporting suicidal distress or providing clear pathways of support.
Population is pre‐registration nursing students.	Population is not pre‐registration nursing students.
Study includes pre‐registration nurses and data can be separated from non‐pre‐registration nursing data.	Study includes pre‐registration nurses but data cannot be separated from non‐pre‐registration nursing data.
Published in English language.	Not published in English language.
Primary research (i.e., quantitative, qualitative or mixed‐methods studies).	Wrong study design (i.e., commentary, discussion paper or letter to editor).
Full text article available on included databases.	Full text article not available.

### 5.5 Charting the Data

All reviewers collectively populated the data extraction template in Covidence, determining variables to extract to answer the research question. This included title; first author's name; year of publication; journal title; grey or peer‐reviewed literature; study design; study aim; country; name and stage of course pre‐registration nurses are enrolled in; and barriers and enablers to preregistration nursing students identifying signs of suicidal distress, engaging with persons with suicidal distress, and providing clear pathways of support. Data was extracted from six articles by one author (RM) with secondary extraction being undertaken by a second member of the research team (AH, SS). Reviewers then met and determined that the three questions (identifying, engaging, treating) could not be answered from the extracted data. The form was refined, with these three variables being replaced with general barriers and enablers to working with people who are suicidal.

To ensure accuracy, text was directly copied from the article to populate each field. To ensure data extracted remained consistent with the research question and purpose, 50% of included articles were subject to independent extraction by two reviewers (RM, SS, AH). Disagreements were resolved through negotiation between the two researchers who extracted the data. This is a deviation from the protocol, which specified that 10 articles would undergo independent extraction by two reviewers. It was agreed by the reviewers that given the number of included articles, a greater number of papers should be extracted by two reviewers to ensure reliability. A summary of extracted data is shown in Table 2.

**TABLE 2:** Summary of included studies.

Author, date, country	Study design and sample	Aim of study	Key findings
Bajracharya et al. (2020), Nepal	Quantitative: descriptive, cross‐sectional design, structured questionnaire conducted with 193 nursing students at a university in Kathmandu.	To assess nursing students' perception towards attempted suicide and to find out the association between perception and selected variables.	This study reveals negative dispositions of nursing students towards attempted suicide. These perceptions related to respondents' feelings, low educational level, social norms that influence these perceptions. The authors conclude that it is vital for nurses to transform their attitudes through training, reflection and clinical psychiatric experience to be more positive and therapeutic towards attempted suicide patients, helping them to eliminate the potential of a future attempt to suicide.
de Albuquerque et al. (2022), Brazil	Quantitative: descriptive and exploratory study using questionnaires with 253 first to fifth‐year nursing students.	To verify attitudes related to suicidal behaviour among nursing students.	Previous experience with suicidal behaviour (ideation, planning or attempt) may have been an important predictor for the nursing students in this survey to present more understanding and less prejudiced attitudes towards suicidal people. Students described difficulty in asking about suicidal behaviour, worried asking may induce someone to go ahead with their plan to suicide; they also felt helpless when faced with an individual with suicidal ideation, a lack of professional preparation to deal with suicidality, and often held conservative and religious attitudes towards suicide. The authors postulate that increasing understanding during the training phase can bring about comprehensive, prejudice‐free nursing care of individuals with suicidal behaviours.
Del Pozo‐Herce et al. (2024), Spain	Qualitative: descriptive phenomenological study using focus groups and reflective narratives in 45 third‐year nursing students in a Spanish university.	To explore the perceptions and experiences of students during clinical simulation (patients at risk of suicide).	The identification and assessment of risks and warning signs during the simulation allowed students to assess risk factors associated with suicide effectively, consolidating their preparedness for real situations. The significance of understanding the underlying motives for suicide was emphasised by participants, highlighting their recognition of the importance of delving into the reasons behind suicidal intentions. Participants also recognised the importance of identifying protective factors, demonstrating a balanced perspective beyond solely focusing on risk factors. The results underscore the crucial role of thorough assessment and the consideration of diverse elements in clinical practice and health professional training.
Fenstermaker (2006), United States	Qualitative: Dissertation using an interpretive phenomenological methodology with 24 Baccalaureate nursing students.	To investigate the lived experience of caring for the suicide attempter from the perspective of the baccalaureate nursing student.	The participants in this dissertation study experienced a sense of awkwardness when they initially approached and caring for suicide attempters. This impacted their confidence. Students also felt that suicide attempters were more difficult to help than medical‐surgical patients. Many participants described how difficult the first experience of caring for a suicide attempter was. They described being unsure of themselves, not knowing what was expected of them, and not knowing what to do. Some wondered if they were qualified to care for a suicide attempter. During the students' initial approaches to their assigned suicide attempters, fear blocked their confidence levels. The students lacked confidence in their abilities to care for suicide attempters. The more the participants thought about caring for their patients, the more their confidence levels diminished.
Ferrara et al. (2021), Italy	Quantitative: Multicentre cross‐sectional survey incorporating a ‘before and after’ design with 314 nursing students.	To explore nursing students' attitudes towards suicidal risk across nursing schools in three Italian universities.	Students who reported direct experience with personal or close ideas of suicide in their lifetime or mental health issues showed more empathy towards the patient, highlighting the importance of direct experiences in reducing the discrimination and increase the inclination towards mental illness and suicide evaluation. Median scores from areas of the survey exploring knowledge about suicide confirmed the absence of fundamental knowledge of the correct evaluation of an individual displaying suicidal ideation.
Goldstein (2017), United States	Quantitative: Dissertation using survey methodology with 16 students enrolled in an accelerated nursing program.	To determine if the two‐hour online suicide prevention gatekeeper training program increases perceived self‐efficacy of suicide assessment and management.	This dissertation found a lack of evidence for education related to suicide prevention in many schools of nursing, and no uniform training for students in suicide assessment. Results from this study suggest that completing the two‐hour online training program produces a statistically significant increase for students in both perceived self‐efficacy and perceived self‐knowledge in assessing and managing suicide. After completing the online training program, participants in the program indicated an increased willingness to directly raise the question of suicidal intent, and had an increased confidence in the ability to have a conversation with someone they suspect is considering suicide.
Hamidi et al. (2024), Canada	Mixed methods: Explanatory sequential design. Quantitative phase using a validated tool and qualitative phase using semi structured interviews with third‐year nursing students (*n* = 130 and *n* = 8) enrolled in a mental health nursing course in an Ontario‐based university.	To explore the effectiveness of a Suicidal Ideation and Assessment of Risk virtual simulation module.	Participants shared that the virtual simulation helped increase their self‐confidence and decrease anxiety‐related feelings when assessing for suicidal ideation. The virtual simulation module on suicidality and assessment of risk was found to be a valuable method to augment critical thinking and knowledge through debriefing, allowing students to reflect and increase their knowledge of suicidality, with the authors postulating that this phase of the simulation helped to build critical thinking skills.
Heyman et al. (2015), United Kingdom	Qualitative: Phenomenological enquiry using focus groups with second year Bachelor of Nursing students (*n* = 10) in a Scottish university.	To understand student experiences of a workshop for suicide intervention skills education (ASIST) through the undergraduate curricula and the impact on learning.	The authors describe the building of confidence, particularly through roleplay, as an important element of the ASIST workshop, suggesting students are better equipped for clinical practice. All participants agreed that they were more confident in suicide intervention. The students also expressed the confidence to move out into practice and use the skills they learned. The authors suggest a staged approach to building suicide intervention skills throughout all years of nursing programs, creating opportunities to develop strong class cohesion to support peer learning of challenging topics, and creating mechanisms to ensure psychological safety when teaching these topics.
Inman et al. (1984), United States	Quantitative: Two multiple‐choice questionnaires administered to 89 students enrolled in a three‐year nursing diploma.	To examine the relationship between knowledge of suicidal lethality factors and the capacity for verbal interview responsiveness to suicidal individuals, and additionally, to determine the association between each of these two factors and selected suicide attitudes and experiences.	The results indicate that those students who believe some potential suicides should be prevented score higher on both knowledge and interviewing skill inventories than either those who think either all or none should be prevented. One explanation for such a finding is that both increased suicide knowledge and interviewing skill are associated with lack of certainty about when or if suicide should be prevented. The authors argue a need for training programs that focus on the acquisition of knowledge on suicidality and specific interviewing techniques to improve responses to suicidality, recognising that knowledge and skill is likely to vary with personal beliefs on suicide prevention.
Kaçan (2023), Turkey	Quantitative: descriptive correlational design conducted with 446 nursing students.	To examine the relationship between the knowledge of nursing students about suicide and their stigmatising attitudes towards people committing suicide, and the factors affecting it.	Students who did not have a family history of psychiatric treatment had a high level of stigmatisation towards those who committed suicide. Male students had low levels of knowledge about suicide and high stigmatisation attitudes, with the authors postulating this may have a negative effect to discourage people from talking about their thoughts about suicide. The stigmatising attitude prevents them from talking about suicidal thoughts, detecting early signs of suicide, or helping people in despair. Students who had a history of receiving professional support had a higher level of knowledge about suicide, having a family member who received psychiatric treatment increased their level of knowledge and positively reduced stigmatising attitudes.
Kerr et al. (2018), United Kingdom	Quantitative: An exploratory study utilising a survey design and repeated measures, conducted with 128 nursing students.	To determine the impact of training developed to help people recognise an individual with suicidal thoughts (SafeTALK) on student nurses' perceived self‐efficacy	The results of the study show that the SafeTALK training had a positive impact on increasing the general self‐efficacy of the participants in the whole sample. Performance of the skills of questioning, active listening and keeping safe will provide mastery experiences especially when supported by constructive feedback and a focus on success in performance of the skills. If these skills are rehearsed in groups, then this provides the opportunity for other students to provide constructive peer feedback. Emotional and affective factors can also influence levels of self‐efficacy regarding suicide prevention. Preparation for coming into direct contact with people that have thoughts and plans for suicide through discussion, normalising the response and the reflective process can help to reduce anxiety and increase self‐efficacy.
Luebbert and Popkess (2015), United States	Quantitative: Two‐group, experimental, post‐test‐only design with random assignment to either a class using a simulation on suicide assessment with a standardised patient (intervention group) or a class using video‐recorded lecture (control), with 34 nursing students at a Midwestern public university.	To develop and test an innovative active learning strategy using simulated standardised patients to determine its effectiveness in teaching suicide assessment skills.	The authors argue that educating nursing students using traditional teaching methods often fails to prepare them adequately to assess patients suicide risk. This study using simulated standardised patients demonstrated a significant difference in student scores of self‐confidence, satisfaction, and student perceptions of the educational practices (active learning, collaboration, and appeal to diverse learning styles) when compared to the lecture format. However, students in the simulation group did not differ from those who received the standard lecture in terms of expectation and knowledge outcomes.
Moraes et al. (2016), Brazil	Quantitative: Cross sectional survey study conducted with 244 nursing students from a rural university in Sao Paulo.	To investigate the suicide‐related attitudes and associated factors among nursing undergraduates.	In this study, female participants held more negative attitudes to suicide, with men and students who had participated in a psychiatric nursing discipline was associated with a greater perception of professional capacity to respond to suicidality. Previous experience with individuals expressing suicidality was not associated with attitudes related to suicidal behaviour. A less “moralistic and condemnatory” attitude to suicidality was found among students who read specific material on suicidality or had suicidal ideation previously.
Oz et al. (2021), Cyprus	Quantitative: Descriptive, cross‐sectional survey study using validated tools with 650 nursing students in a university in Northern Cyprus.	To assess the stigmatising attitudes of nursing students towards individuals who had made suicide attempts as well as these students' attitudes towards death.	Students without any acquaintances who had attempted suicide had more positive attitudes towards death, with a neutral attitude towards death – in the framework of this study, a neutral and approach acceptance is where an individual believes death is a part of life and accepts life with this fact. Statistically significant differences between the stigmatising attitudes of different students towards individuals who had attempted suicide, which the authors concede may cause nursing students to overlook or ignore help‐seeking behaviours from individuals experiencing suicidality. In this study, female students had higher levels of stigma towards suicide compared to male students. However, students who had relatives who had completed suicide where more likely to have stigmatising attitudes.
Pederson (1993), United States	Quantitative: a post‐test control group design within the con‐text of a larger investigation of the effectiveness of structured controversy on knowledge, attitude, and skills in discussing controversial issues.	To compare the effectiveness of structured controversy with lecture on students' attitudes towards providing nursing care for patients who are suicidal.	Students who used structured controversy (an interactive teaching strategy in which students, in small groups, argue both for and against a position and then attempt to reach consensus on the given position) were significantly more positive than students who just attended a lecture, with the authors arguing that student feelings of stress and guilt were influenced in a more positive manner by participating in the structured controversy than listening to a lecture. Attitude was noted in this study to be a strong determinant of student intentions to provide care to individuals expressing suicidality.
Poreddi et al. (2021), India	Quantitative: Descriptive, cross‐sectional survey study conducted with 223 undergraduate nursing students from a university in southern India.	To evaluate nursing students' attitudes towards suicide and their role in suicide prevention.	In this study, age and education significantly differ in participants' attitudes towards suicidal behaviour in various domains. Students who have completed theory and clinical hours in mental health nursing hold favourable attitudes towards suicidal behaviours. This study's findings showed that most nursing students had favourable attitudes towards patients with suicidal behaviours and suicide prevention.
Pullen et al. (2016), United States	Mixed methods: Study including both qualitative and quantitative survey data of 150 nursing students enrolled in psychiatric nursing in a college in Montana.	To describe baccalaureate nursing students' responses to an evidence‐based practice program implemented in one undergraduate college of nursing in Montana.	Quantitative data showed a statistically significant increase in both the understanding and comfort level with suicide prevention after the program. Qualitative data showed that students felt more capable intervening with persons at risk for suicide after the program. Overall, this study showed increased feelings of capability and self‐perceived knowledge base on completion of the course.
Quemada‐Gonzalez et al. (2024), Spain	Qualitative: Qualitative descriptive study using a questionnaire of three open‐ended questions administered to 72 nursing students after completion of simulated scenarios.	To explore nursing students' perceptions, thoughts, and emotions about their performance in dealing with risk for suicidal behaviour through simulated scenarios.	This study indicates a tendency for students to experience negative emotions when dealing with suicidality. The emotions reported by the students in this study coincide in part with the results of other research conducted with graduate nurses being identified as a barrier in the care provided. Three themes were evident after content analysis of the written responses: emotions experienced during the simulation, which were largely negative and included anxiety and insecurity, self‐criticism of performance during the simulation, and student evaluation of the learning experience. Students described the experience of simulation as positive for their professional and personal learning.
Rebair (2019), United Kingdom	Qualitative: Dissertation using Grounded Theory using focus groups, semi‐structured interviews and field notes with student nurses (*n* = 16) and individuals with suicidal ideation (*n* = 9).	To gain understanding of student nurses and suicidal individuals‚ and to experience of engaging in conversations about suicide.	In this dissertation, many statements from student nurses collectively demonstrated judgement and a lack of understanding of the suicidal person. A study theme of “Lost in translation” referred to the consequences of acting from the limitations of self‐belief and establishing belief in others. This created so‐called limiting spaces. In limiting spaces, the student nurse was unable to see the other as they are too focussed on self or determining truth, rather than hearing a story and being with the suicidal person. Occupying these spaces was limiting in relation to students feeling unable to enter conversations about suicide with another and thus created limitations within the pivotal encounter.
Scheckel and Nelson (2014), United States	Qualitative: Hermeneutic phenomenological study using in‐depth, unstructured interviews with 12 senior nursing students in a Midwestern university.	To obtain insights into the basic preparation of students in the care of suicidal persons to inform pedagogical approaches pertaining to suicide and improve the nursing care for these individuals.	When describing their experiences of assessing suicidal ideation, some participants remarked suicide was a taboo topic, and hence, it would be difficult asking patients about suicidal ideation. Not all participants described being able to assess suicide risk by listening to and hearing patients talk about their suicidal tendencies. A few participants reported times when patients were less forthcoming or silent about their suicidal tendencies. In these cases, participants remained uncomfortable with patient interactions, often not knowing what to do or say next.
Sun et al. (2011), Taiwan	Quantitative: Quasi‐experimental study of 174 nursing students, comparing the efficacy of a 4‐h suicidal education program with a control group who did not attend the program.	To investigate the learning outcomes of a suicide education programme for second‐year student nurses in Taiwan.	Participants in the experimental group held more positive attitudes towards the acceptance of suicidal behaviours and were non‐judgmental in their morality. Further, they showed more positive attitudes towards the provision of professional care and believed that people who attempt suicide are communicating their psychic pain. Participants in the experimental group also held more positive beliefs about people who attempt suicide than the control group did, including more positive attitudes towards the provision of professional care and a belief that individuals who express suicidality are “… communicating their psychic pain,” (p. 837).
Sun et al. (2019), Taiwan	Qualitative: Grounded Theory using semi‐structured interviews with 22 nursing students who had provided care to individuals with suicidal ideation.	To develop a theory to guide nursing students when caring for patients with suicidal tendencies on their psychiatric clinical practicum.	Some nursing students had a negative mindset towards suicidal patients, for example, they saw it as ‘stupid’ or ‘irresponsible’ behaviour. When nursing student participants perceived they had a positive mindset towards suicidal patients, for example, they wanted to care for patients, and they had come to accept them. When patients gave the students negative feedback, for example, some patients rejected them and told them to go away, a few patients were unwilling to talk about their experiences, some nursing student participants perceived they lacked the competencies required to provide therapeutic care to these challenging patients, such as difficulty in changing patients suicidal ideation.
Sun et al. (2020), Taiwan	Qualitative: Grounded Theory using semi‐structured interviews and constant comparative analysis with 22 nursing students who had cared for patients with suicidal ideation for at least 5 days during their clinical placement.	To explore the psychological processes experienced by nursing students caring for suicidal patients during their first psychiatric clinical practicum.	Students were apprehensive in case the patients would attempt suicide and, perhaps, die by suicide during their psychiatric clinical practicum. They worried in case they would not be able to deal with a suicidal situation. Students were afraid in case they might say something that made the patients feel sad because, they perceived suicidal patients to be very sensitive. Moreover, they were afraid that if they spoke too straightforwardly this might hurt the patients' feelings and then they would feel guilty. Students wanted to provide best possible care for patients. However, no matter how they tried, the patients did not change their negative thinking and suicidal ideations. The participants alluded that they felt a sense of frustration and powerlessness.
Vedana et al. (2018), Brazil	Qualitative: Grounded Theory using semi‐structured interviews with 30 nursing students.	To gain an understanding of the meanings of suicidal behaviour for a particular group of nurses.	The participants perceived suicidal behaviour as negatively distinct from other health conditions, primarily because it is perceived as a ‘self‐inflicted’ condition, often viewing the perceived motivations behind suicide as conflicting with the life‐saving principles and ethics of the healthcare profession. Participants also acknowledged the existence of personal difficulties (in terms of their empathy for a person at risk of suicide) and the wide‐spread nature of the stigma, prejudice, and discrimination to which suicide is subject. The comprehension of such sensitive issues seemed to be greater in participants with some sort of personal experience connected with suicidal behaviour. Participants attested to experiencing conflict between their personal beliefs and the professional conduct required of them in care situations.
Vedana and Zanetti (2019), Brazil	Quantitative: Cross‐sectional survey, using validated instruments, with 111 final‐year nursing students.	To investigate attitudes related to suicidal behaviour and associated factors.	Most intense negative feelings towards people with suicidal behaviour were correlated with lower perceptions of professional competence, increased moralistic or condemnatory attitudes, and younger age. The negative feelings and attitudes towards suicidal behaviour may also impair the quality of care provided by health professionals to patients who are feeling suicidal or have attempted suicide. At the end of the nursing course, students had a less negative attitude and a greater perception of their own competence in dealing with suicidal behaviour. The participation in suicide‐related scientific events, courses or lectures were associated with more moralistic or condemnatory attitudes towards suicidal behaviour.
Vedana et al. (2020), Portugal	Quantitative: Cross‐sectional survey of 351 nursing students conducted in a Portuguese higher education institution.	To investigate the attitudes of nursing students towards suicide and associated factors.	Students nearing the end of their nursing course and those who were older had more positive attitudes towards in individuals with suicidal ideation, and greater self‐perception of their professional competence. Negative feelings towards the person with suicidal behaviour were more intense when the students had lower self‐perception of professional competence. Unexpectedly, students who had attended events, courses or lectures about suicide held more moralistic or condemnatory attitudes towards suicidality.
Vedana et al. (2022), Portugal	Qualitative: Grounded Theory using individual, semi‐structured interviews with 13 undergraduate nursing students.	To understand the meanings of suicidal behaviour for Portuguese undergraduate students.	The findings indicated that suicidal behaviour, classified according to the individual beliefs and judgements of the participants, presented a significant barrier to the delivery of care and was complex and multifaceted care phenomenon. Participants were often reluctant to discuss suicidality and seemingly wanting to distance themselves from the care of persons exhibiting suicidal behaviour, and to avoid professional engagements relating to suicide prevention. Suicidality was discussed as a neglected risk, believed to be due to its characteristics of hidden emotional states and social issues that impede preventing suicidality, for example non‐recognition of risk and inappropriate communication.
Yoo et al. (2021), South Korea	Qualitative: Focus group interviews conducted with 36 nursing students who had participated in suicide prevention volunteer activities for 4 months.	To explore the experience of nursing students who participated in suicide prevention volunteer activities.	Students experienced changes in their attitudes and beliefs towards suicide issues through participation in suicide prevention volunteer activities. Students had a sense of responsibility and obligation towards suicide prevention activities, with suicide ‘gatekeeper’ education, which, along with suicide prevention activities, was felt to have improved knowledge and competence in suicide prevention. By preparing for and conducting suicide prevention activities, students were better able to understand mental ill health, with stigma against mental illness decreasing, and students were said to develop an interest in mental health nursing.

### 5.6 Collating, Summarising and Reporting the Results

Extracted data was exported from Covidence to Microsoft Excel (version 1808) and displayed in table format, enabling a visual summary of the study characteristics. Using a deductive approach, three members of the research team (SS, BH, RM) independently familiarised themselves with the data and categorised it according to the three subquestions: What are the barriers and enablers to pre‐registration nursing students (1) identifying, (2) engaging with and (3) treating individuals experiencing suicidal thoughts? Categorised data were discussed and confirmed with another member of the research team (AS). Three members of the research team (RM, SS, BH) once again independently familiarised themselves with the extracted data before meeting to discuss and collaboratively identify themes. Themes have been presented in a narrative summary.

#### 5.6.1 Barriers and Enablers to Identifying Suicide Risk

Identifying suicide risk encompasses recognising risk, screening and assessing for risk and using clinical judgement to develop a formulation of risk. This requires student nurses to interact with the person at risk and to discuss the topic of suicide. However, some student nurses have reported reluctance to discuss suicide and a tendency to distance themselves from the care of persons at risk of suicide (Vedana et al. 2022). This avoidance has been attributed to individual beliefs and judgements (Vedana et al. 2018), stigmatising attitudes (Kaçan 2023), difficulties talking about a topic they consider as taboo (Scheckel and Nelson 2014) and fear about saying the wrong thing (Rebair 2019) or saying something that will prompt a suicide attempt (Sun et al. 2019). Furthermore, some student nurses described how reading patient notes prior to conducting a suicide assessment resulted in fear of initiating an interaction:

Reading from his chart, he was very angry and aggressive toward a [family member] who didn't feel safe being alone with him. I was nervous to be his nurse and to be asking him those questions [about suicidal ideation] and doing [suicide] assessments on him. (Scheckel and Nelson 2014)

Hamidi et al. (2024) suggest that nursing students lack knowledge and clinical experience in the assessment of suicide risk. Consequently, student nurses experience challenges when attempting to conduct assessments, with some reporting difficulties assessing healthcare consumers who are silent or not forthcoming about their suicidal thinking (Scheckel and Nelson 2014), and others reporting difficulties in recognising symptoms of suicide (Kaçan 2023) and internal suffering (Vedana et al. 2022).

Various educational interventions have shown promise in enabling nursing students to identify suicide risk. For example, after completing a 2‐h online program focusing on screening for suicide, student nurses showed a statistically significant increase in perceived self‐efficacy and perceived self‐knowledge in suicide assessment (Goldstein 2017). It has been suggested that non‐traditional teaching methods are required to adequately prepare nursing students to assess suicide risk (Luebbert and Popkess 2015), with simulation‐based education being found to be effective in preparing student nurses for practice (Hamidi et al. 2024). This approach improves knowledge, self‐confidence, critical thinking and decreases anxiety (Del Pozo‐Herce et al. 2024; Hamidi et al. 2024; Luebbert and Popkess 2015). Notably, student nurses who participated in simulation‐based education developed a profound appreciation for the significance of establishing a solid initial connection when conducting an assessment (Del Pozo‐Herce et al. 2024). In addition to simulation‐based education, extra‐curricular activity experiences of suicide prevention whereby students promote suicide prevention to the public and work with older people in the community to promote their mental health improved students' skills and confidence in relationship building and ability to respond to suicide crises (Yoo et al. 2021).

#### 5.6.2 Barriers and Enablers to Actively Engaging With People at Risk of Suicide

Actively engaging with healthcare consumers at risk of suicide involves ensuring that all of those who have been identified as being at risk for suicide are active participants in their own care and connected with collaborative care and supports (Zero Suicide 2024b). This means that student nurses need to engage with healthcare consumers at risk of suicide and work in partnership with them. However, student nurses often felt that they had insufficient knowledge to be able to care for and meet the needs of the healthcare consumer experiencing suicidal thoughts, which resulted in the student nurse not engaging with the person (Fenstermaker 2006; Kaçan 2023; Sun et al. 2019; Vedana et al. 2018). Low levels of knowledge also led to stigmatising attitudes and the inability to talk and engage with healthcare consumers about their suicidal ideation (Kaçan 2023). Decreased or poor attitudes also led to an inability to engage with healthcare consumers experiencing suicidal ideation (Vedana et al. 2020; Vedana et al. 2018; Vedana and Zanetti 2019).

By gaining knowledge, the ability to engage with people experiencing suicidal thoughts, attitudes and empathy towards healthcare consumers expressing suicidality improved (Fenstermaker 2006; Kerr et al. 2018; Sun et al. 2019; Sun et al. 2020). In their 2018 study, Kerr et al. described that by providing education that involves practising skills of listening, questioning and keeping safe, students' perceived knowledge base improved and made them more confident in engaging with healthcare consumers experiencing suicidal ideation and behaviours. Students also had a fear where they needed to focus on themselves and their own safety rather than engaging and being with the suicidal person (Rebair 2019; Sun et al. 2020). Students were worried about looking after people, and this disrupted their perceived ability to provide care (Fenstermaker 2006; Rebair 2019; Scheckel and Nelson 2014; Sun et al. 2020).

Because of fear and worry about individuals expressing suicidality, students often did not know what to say or did not want to say the wrong thing. This led to poor engagement with people in their care and tied into their perceived lack of knowledge about the healthcare consumer experiencing suicidal ideation (Fenstermaker 2006; Scheckel and Nelson 2014; Sun et al. 2020). Student confidence made an impact on whether they would engage with healthcare consumers experiencing suicidal ideation; if the person spoke to the student and answered their questions freely, the student felt like they could engage with the person and provide the care that they needed to (Fenstermaker 2006; Scheckel and Nelson 2014; Sun et al. 2020). However, if the person did not engage with the student or did not answer their questions, or even asked them to go away, then the student did not want to provide care, lost confidence and found it difficult (Fenstermaker 2006; Kaçan 2023; Rebair 2019; Sun et al. 2020; Vedana et al. 2018). The more the student engaged through their placement, the greater the confidence and then the more likely they were to continue engaging with healthcare consumers (Fenstermaker 2006).

#### 5.6.3 Barriers and Enablers to Providing Treatment to People at Risk of Suicide

Few studies explored the barriers and enablers to providing treatment to healthcare consumers at risk of suicide by pre‐registration nurses. Pre‐registration nurses appreciated teaching strategies by educators which nurtured and supported them (Sun et al. 2019), as well as workshops and roleplays which specifically equipped them with skills to feel more confident (Heyman et al. 2015), comfortable, capable (Pullen et al. 2016), and overall prepared (Sun et al. 2019) to engage with healthcare consumers at risk of suicide before beginning their practicum. Knowledge gained during practicum gleaned indirectly from multidisciplinary meetings in the clinical environment about individual healthcare consumers helped students to more deeply and holistically understand individual experiences and how to provide effective support (Sun et al. 2019). This led some students to appreciate that suicidal ideation was an expression of ‘psychic pain’ (Sun et al. 2011). Students felt more comfortable providing nursing care to healthcare consumers with suicidal ideation who made disclosures, had support from other people and cooperated with nursing care (Sun et al. 2019). No barriers to providing treatment to people at risk of suicide were identified.

#### 5.6.4 Individual Perspectives of Student Nurses

While theming the data, it became apparent that not all data could be categorised according to the three elements of the Zero Suicide Framework (identify, engage, treat). These three categories specify actions that all student nurses can take; however, they do not consider the unique perspectives and experiences of the individual student nurse. A student nurse's ability to identify, engage and treat a person at risk of suicide is influenced by their own perspective on suicide, along with their own lived and living experience of mental health conditions, which was a present theme in the literature included in this review. Therefore, this category has two sub‐themes: (1) Interpersonal attitudes and beliefs as a factor influencing student nurses caring for suicidal distress, and (2) Lived and living experience as a factor influencing student nurses caring for suicidal distress.

##### 5.6.4.1 Interpersonal Attitudes and Beliefs as a Factor Influencing Student Nurses Caring for Suicidal Distress

Negative attitudes of student nurses towards suicide impact the care provided to healthcare consumers with suicidal distress (Sun et al. 2011), including decreased professional competence, increased moralistic or condemnatory attitudes (Bajracharya et al. 2020), and decreased willingness to provide nursing care (Vedana et al. 2018). Preventing suicide requires student nurses who are intervening to possess an empathic understanding and attitude towards the healthcare consumer experiencing suicidal distress (Vedana et al. 2020). Negative attitudes have been attributed to student nurses' feelings, educational level, age and social norms (Bajracharya et al. 2020; Poreddi et al. 2021). Another explanation may be that some student nurses perceive suicidal behaviour as negatively distinct from other health conditions, describing it as ‘self‐inflicted’ and at odds with the lifesaving approach they have learned to take towards healthcare (Vedana et al. 2018).

Education offers a promising approach to fostering more understanding and less stigmatizing attitudes towards suicide among nursing students (Kaçan 2023; Pederson 1993; Sun et al. 2011; Vedana and Zanetti 2019). However, while it has been suggested that using interactive teaching strategies results in more positive attitudes towards suicide (Pederson 1993), some studies have found that simply engaging with reading materials on suicide can lead to better attitudes (Vedana et al. 2020). Supported opportunities for students to interact with healthcare consumers who are experiencing suicidal distress, for example, clinical placement and volunteering, have also led to positive changes in attitude and beliefs and increased empathy (Pederson 1993; Yoo et al. 2021). An important consideration for these supported opportunities is that students have been found to benefit from the assistance of experienced clinicians, for example, clinical teachers and nurses, to support them to release and resolve negative emotions (Sun et al. 2020).

##### 5.6.4.2 Lived and Living Experience as a Factor Influencing Student Nurses Caring for Suicidal Distress

Nursing students who have had personal experiences with suicide including ideation, planning and attempts tend to exhibit more understanding and less judgmental and stigmatizing attitudes towards healthcare consumers at risk of suicide (de Albuquerque et al. 2022; Kaçan 2023; Moraes et al. 2016; Vedana et al. 2020). Furthermore, those who had a personal experience connected with suicidal behavior had greater empathy towards those experiencing suicidal distress and had greater comprehension of the stigma, prejudice and discrimination associated with stigma.

I had a lot of stigma, you know? The first time some‐one said they were going to kill themselves, I said: so kill yourself! (…) and my ex‐boyfriend threw himself in front of a car, right in front of me. (…) today I'm much more open. I have a much better view. (Vedana et al. 2018, 10)

In addition, those who had received professional support for suicide have been found to have a higher level of knowledge about suicide (Kaçan 2023). Having a family member with a history of receiving psychiatric treatment may has been shown to increase knowledge and decrease stigmatising attitudes towards suicide (Kaçan 2023). However, in contrast, it has also been found that nursing students who do not have any acquaintances who have attempted suicide have more neutral and positive attitudes towards death (Oz et al. 2021).

## 6 Discussion

This review aimed to identify research gaps by mapping what is known about barriers and enablers to pre‐registration nursing students identifying signs of suicidal distress and providing clear pathways of support. As a result of this scoping review, several barriers and enablers were identified, which are summarised in Table 3.

**TABLE 3:** Summary of barriers and enablers described in included studies.

Domain	Barriers	Enablers	Bajracharya et al. (2020)	de Albuquerque et al. (2022)	Del Pozo‐Herce et al. (2024)	Fenstermaker (2006)	Ferrara et al. (2021)	Goldstein (2017)	Hamidi et al. (2024)	Heyman et al. (2015)	Inman et al. (1984)	Kaçan (2023)	Kerr et al. (2018)	Luebbert and Popkess (2015)	Moraes et al. (2016)	Oz et al. (2021)	Pederson (1993)	Poreddi et al. (2021)	Pullen et al. (2016)	Quemada‐Gonzalez et al. (2024)	Rebair (2019)	Scheckel and Nelson (2014)	Sun et al. (2019)	Sun et al. (2020)	Sun et al. (2011)	Vedana et al. (2020)	Vedana et al. (2022)	Vedana et al. (2018)	Vedana and Zanetti (2019)	Yoo et al. (2021)
Knowledge about suicide	Judgement and lack of understanding of the suicidal person.		✔			✔					✔			✔	✔	✔		✔	✔		✔	✔	✔	✔	✔	✔	✔	✔	✔	
	Low levels of understanding of suicidality.		✔			✔	✔	✔			✔	✔	✔	✔	✔	✔			✔		✔	✔	✔	✔	✔	✔	✔	✔	✔	
		Exposure to individuals expressing suicidality increased knowledge.	✔	✔		✔		✔	✔			✔	✔		✔			✔	✔		✔		✔	✔						✔
		History of receiving professional support leads to higher levels of knowledge.					✔					✔				✔														
		Knowledge developed through exposure to different viewpoints and peer perspectives.							✔	✔							✔													✔
Beliefs about suicide	Reluctance to discuss or assess suicidality due to stigmatising beliefs.		✔								✔	✔	✔	✔				✔			✔	✔	✔		✔	✔	✔	✔	✔	✔
	Conflict between personal beliefs and professional responsibilities.		✔									✔				✔	✔	✔				✔	✔			✔		✔	✔	✔
		Attendance at educational programs improved beliefs about suicide.	✔		✔			✔	✔	✔	✔		✔	✔				✔	✔				✔		✔		✔			
Attitudes (towards individual and self)	Lack of confidence in providing care for individuals expressing suicidality.			✔		✔													✔	✔	✔	✔	✔	✔	✔	✔	✔	✔	✔	✔
	Fear of providing care for individuals expressing suicidality.					✔							✔							✔	✔	✔	✔	✔			✔			
		Use of simulation or roleplay during nursing education to increase confidence.			✔				✔	✔			✔	✔					✔	✔									✔	
		Lived experience of suicidality or suicide ideation improves attitude towards suicidality.		✔			✔								✔	✔											✔			✔
Emotions (own emotions in the moment)	Fear of ‘saying the wrong thing’ invoking suicide attempts.			✔		✔																✔		✔						
	Experience of negative emotions when working with individuals expressing suicidality.																			✔				✔						
		Simulation decreasing negative emotions.			✔				✔	✔										✔										
Skills	Perceived lack of skills to begin and maintain conversations assessing suicidality.			✔		✔													✔	✔	✔	✔	✔	✔				✔		✔
		Skills increases shown by the recipients of brief training or during nursing course, including in assessment of risk.			✔			✔	✔	✔	✔		✔	✔				✔	✔	✔					✔	✔		✔	✔	✔

The predominant barrier noted in the literature was that of judgment, stigma and a general lack of understanding of individuals expressing suicidality and suicide itself. In many of the studies included in this review, suicide was considered ‘at odds’ with medical and nursing ideals of preserving life, and given the diversity of geographic regions in this study, many of the included papers describe cultural and religious beliefs on suicide being expressed by student nurses. Existing research notes that individuals who have been hospitalized following a suicide attempt also experience this stigma, with Shih et al. (2023) conducting a qualitative interview study with nine individuals hospitalized in mental health settings after surviving a suicide attempt, finding that six of the participants had experienced st stigmatized views from healthcare providers, including ‘arrogant’ and ‘patronising’ attitudes from nurses, and a feeling of being treated as a diagnosis rather than an individual. Stigma from healthcare workers is especially concerning; literature from the World Health Organization's Mental Health Surveys of 55,302 participants indicates that 7% of participants described stigma as a reason for not seeking treatment, with suicide attempters being the most likely among those surveyed to seek treatment. This indicates that there is a strong need to address this stigma among nursing students to ensure that future clinicians can engage individuals expressing suicidality in effective treatment.

This lack of understanding and stigma was described in the literature included in this scoping review as leading to situations where nursing students were reluctant to assess suicidality, lacked the confidence to raise suicidal ideation, or were fearful of suicidality. It is clear that these situations must be addressed to improve the skills of suicide assessment by pre‐registration nursing students. A modified Delphi study that surveyed an expert panel of 60 nursing faculty, psychiatric nursing faculty, nurse administrators and nurse clinicians was conducted to determine competencies required for students (Kotowski and Roye 2017). These competencies had high agreement around those involving assessment, including risk assessment in a variety of settings, and the importance of improving the knowledge of suicide to prevent situations where reluctance or a lack of confidence prevented assessment of suicidality. As found in this scoping review, a lack of knowledge of suicidality was a key barrier to students assessing for suicidality, and this barrier may persist into the transition to clinical practice. Further, a systematic review of the Zero Suicide Framework and other suicide prevention programs conducted in Australia indicated that continued education was an important factor to maintain skills in addressing suicidality and assessing risk (Dabkowski and Porter 2021).

Furthermore, four papers described situations where student nurses felt that raising suicidal ideation would lead to individuals acting on this, therefore invoking a suicide attempt (de Albuquerque et al. 2022; Fenstermaker 2006; Scheckel and Nelson 2014; Sun et al. 2020). This perception has been a long‐held view in the media, where news reports have withheld information on suicide for fear that ‘copycat’ behaviour will occur (Gualtieri et al. 2024; Machlin et al. 2012). However, a large systematic review and meta‐analysis on the risks of asking about suicidality and harm found that consistent, emphatic questioning did not invoke harm from either psychological distress, suicide‐related behaviours, or non‐suicidal self‐injury (Polihronis et al. 2022). These findings are supported by Dazzi et al. (2014), who found no statistically significant increase in suicidal ideation among participants asked about suicidality, finding that asking about suicidal ideation may reduce suicidality among treatment‐seeking populations. In tandem, these studies also indicate the importance of nursing students holding sufficient knowledge to address suicidality; this knowledge should also include correction of misconceptions around suicide assessment.

Despite these barriers, several enablers were noted to improve the ability for student and novice nurses to address suicidality with individuals using healthcare services. Primarily, the studies included in this review noted that exposure to individuals expressing suicidality, through clinical placement, volunteer programs or educational endeavours, improved attitudes among students, increased knowledge and ultimately, reduced barriers to assessing and discussing suicidality with healthcare consumers. This finding highlights the importance of providing students with mental health clinical placements during their pre‐registration training, both to provide them with exposure to situations where mental ill health may occur and to allow them to build skills in assessing and intervening in mental health crises. This importance has been underlined by authors such as Happell (2008), who described clinical experience for students in mental health as improving attitudes towards mental ill health and mental health nursing generally, and Perlman et al. (2022), whose pre‐ and post‐test study of 210 Australian nursing students found that exposure to this environment resulted in significant differences in therapeutic relationship skills.

A lack of skill in initiating conversations around suicidality, assessing and ‘knowing what to say’ was noted among several studies included in this review. Potentially, this finding leads to the disengagement of individuals when they are most likely to describe suicidality, resulting in lost opportunities to intervene and prevent individuals from acting on suicidal ideation. However, a strong enabler to improving this lack of skill was noted to be simulation‐based education. High‐fidelity simulation and simulated patient simulation have barriers to implementation, such as cost and the provision of appropriately trained clinical actors (Lee et al. 2018). However, promising results have been found in other approaches, such as virtual simulation. For example, a virtual simulation program conducted with 105 nurse educators, family nurse practitioners and nurse anesthesia students in the United States found that virtual simulation was a valuable learning tool, allowing students to practice difficult conversations in a controlled environment (Perez et al. 2022). Similar results were found with mental health nursing students (Bahadur et al. 2024); however, traditional high‐fidelity simulation should not be discounted as an effective means to improve knowledge on suicidality, reduce stigma and enhance willingness to intervene and assess suicidal ideation (Heyman et al. 2015; Magerman et al. 2022). In our scoping review, simulation and role play also reduced the experience of negative emotions when working with individuals who express suicidal ideation.

Finally, exposure to content that explores suicidality, such as mental health nursing content in courses, was found in the literature included in our scoping review to improve attitudes and confidence to work with individuals expressing suicidality. Likewise, short courses were also noted to improve attitudes, willingness to work with individuals who express suicidality and reduce stigma.

### 6.1 Application of These Results to the Zero Suicide Framework

Two unanticipated findings from this review have implications for the ZSF. The first is that personal attitudes and beliefs influence student nurses caring for persons with suicidal distress. The ‘train’ element of the ZSF specifies that ‘Employees are assessed for the beliefs, training and skills needed to care for individuals at risk of suicide’. A closer examination of the ZSF workforce survey provided for undertaking the training element shows that none of the questions address the personal beliefs of employees. This is a surprising observation because there is a complex relationship between clinician knowledge of and attitudes towards caring for people at risk of suicide (Boukouvalas et al. 2020), influencing how they interact with persons experiencing suicidal distress, potentially affecting the quality of care provided (Bahamón et al. 2024). Educators should consider supplementing the ZSF workforce survey with questions that specifically address the beliefs, attitudes and lived and living experience of staff to inform local training and implementation efforts. This point also links to the second unanticipated finding that lived and living experience are factors influencing student nurses caring for suicidal distress because both personal and professional experiences with suicide also positively impact attitudes and confidence (Boukouvalas et al. 2020).

The ‘lead’ element of the ZSF includes specific mention of lived experience; however, this only considers the lived experience of persons receiving care, not those providing the care. Research shows that lived experiences of mental health problems among mental health professionals are common, yet the disclosure of such experiences in the workplaces is less so because of stigma, exclusion and negative impacts on careers (Zamir et al. 2022). These nurse ‘prosumers’, a term combining the words ‘professional’ and ‘consumer’ (Manos 1992), cite personal experience of mental illness as a motivator and an aspect of their identity that enhances nursing expertise (Oates et al. 2017). This extends beyond nursing to other professions where those who have experienced depression were less likely to hold beliefs stigmatizing towards patients and show increased empathy and optimism about recovery, enhancing their clinical work, allowing improved empathy, more nuanced questions and more personally tailored interventions (Taylor 2024). This suggests that the ZSF should specifically address the personal beliefs and attitudes of clinicians and incorporate their lived and living experiences. In addition, educators should consider supplementing the ZSF workforce survey with questions that specifically address the beliefs, attitudes and lived and living experiences of staff to inform local training and implementation efforts.

## 7 Strengths and Limitations of the Work

To our knowledge, this is the first scoping review that explores the barriers and enablers to pre‐registration students exploring and assessing suicidality; however, like all reviews of this kind, there are limitations to consider when interpreting our findings. A substantial limitation of this scoping review was the diversity of literature, particularly regarding the lack of evaluation methods in many of the studies that describe improvements in attitudes and skills after educational programs had been administered. When using a scoping review methodology, quality appraisal of the included literature is not conducted, meaning that our study did not seek to identify limitations of individual studies included in the review. As indicated by the JBI guidance for producing scoping reviews (2024), the aim of a scoping review is to map components of evidence rather than answer precise clinical questions. This specification should be recognised when considering methodological limitations in the evaluation processes of the included studies.

During the search, studies not published in the English language or in peer‐reviewed journals may have been missed during the search process. However, the goal of this scoping review was to identify barriers and enablers rather than critique the quality of methodologies in the included literature. We also suggest studies are conducted to determine whether simulation and education programs addressing the barriers outlined in this review have the fidelity to maintain knowledge from the pre‐registration setting to clinical practice. As far as we can determine, this scoping review is the first application of the structure of the ZSF to pre‐registration clinicians, including nurses. This review also contributes to the small existing literature about the relationship of nursing to the ZSF and suggests revision of the ZSF to address staff attitudes and beliefs and lived and living experience.

## 8 Conclusion

Nurses are in an ideal position to assess suicidality and suicidal ideation among healthcare consumers, and preparation for this needs to begin at the pre‐registration level. Our scoping review has identified several barriers and enablers to pre‐registration nursing students' ability to provide safe care for healthcare consumers expressing suicidality, including a lack of knowledge about suicide, stigma, a perceived lack of skills and a feeling that saying the ‘wrong thing’ when working with individuals expressing suicidal ideation may invoke suicide attempts. Several enablers were found that build confidence and skills to address suicidality, including lived experience, exposure to individuals with suicidal ideation and mental ill health more generally, and education and simulation to improve knowledge and refine skills. We recommend further research among pre‐registration nursing students to formulate strategies to address these barriers and enhance enablers to improve the care provided to healthcare consumers who express suicidality.

## Author Contributions

All authors have agreed on the final version and meet at least one of the following criteria (recommended by the ICMJE): substantial contributions to conception and design, acquisition of data, or analysis and interpretation of data; drafting the article or revising it critically for important intellectual content.

## Conflicts of Interest

The authors declare no conflicts of interest.

## Supporting information


**Data S1:** jan70274‐sup‐0001‐DataS1.docx.

## Data Availability

The authors have nothing to report.
