# Supplementary material for: Barriers and Enablers to Pre‐Registration Nurses Providing Safe Care for Individuals Experiencing Suicidal Distress: A Scoping Review
Source: J Adv Nurs. 2025 Sep 30;82(6):5638–58. doi: 10.1111/jan.70274 (PMC13176729; doi:10.1111/jan.70274)
Supplement: Supplementary file 1 — Data S1: jan70274‐sup‐0001‐DataS1.docx. [file JAN-82-5638-s001.docx]

**Search Strategy Scopus**

(TITLE-ABS-KEY(suicid* OR parasuicid* OR para-suicid* OR overdos* OR nssi OR dsh OR selfinflict* OR selfharm* OR sdv OR selfmutilat* OR selfpoison* OR selfinjur* OR automutilat* OR hanging OR ligatur* OR strangulat*) AND TITLE-ABS-KEY((student* or pre-registration*) W/4 nurs*))

**Search Strategy Cinahl**


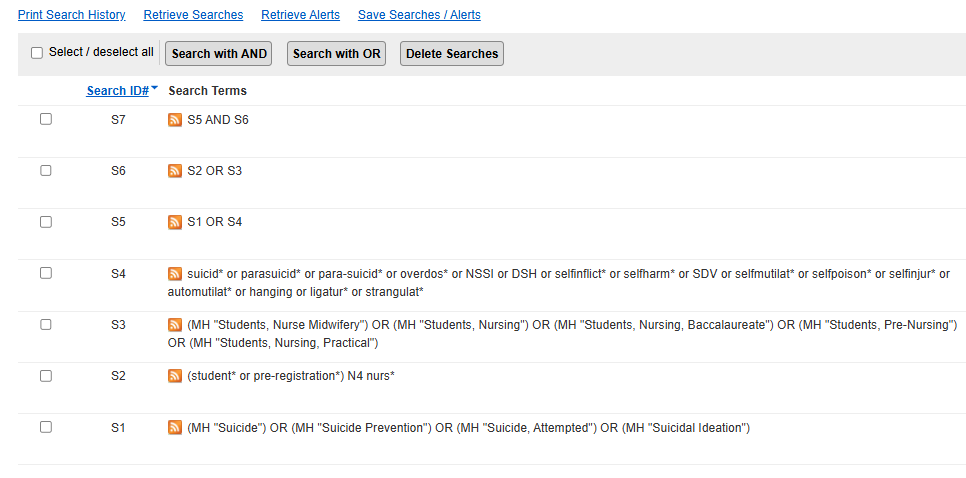


Search strategy psycinfo


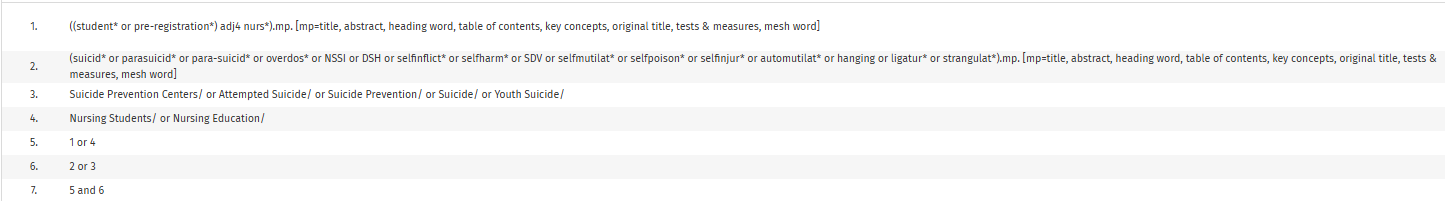


Search Strategy Emcare


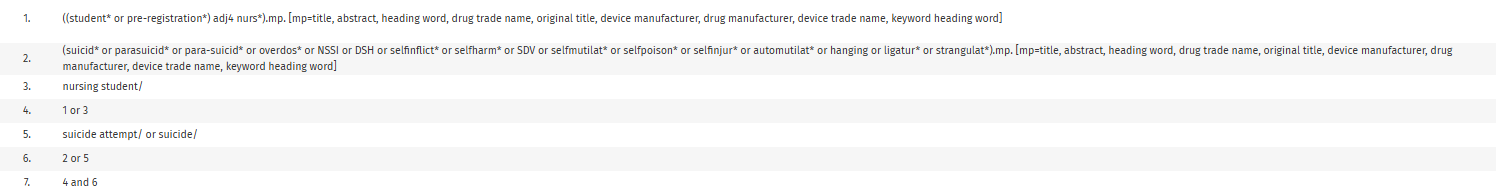


Search strategy Medline


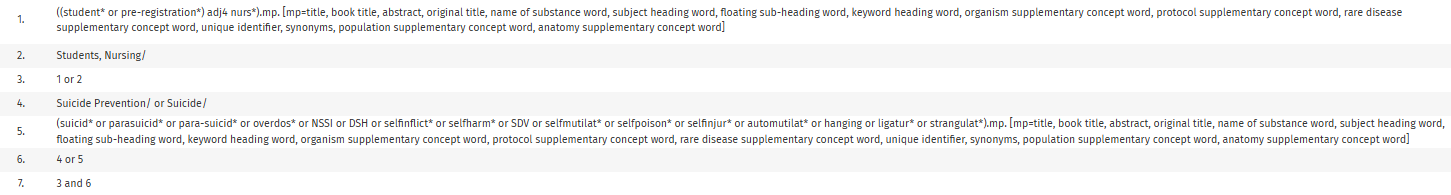


**Search strategy Eric**

(suicid* OR parasuicid* OR para-suicid* OR overdos* OR nssi OR dsh OR selfinflict* OR selfharm* OR sdv OR selfmutilat* OR selfpoison* OR selfinjur* OR automutilat* OR hanging OR ligatur* OR strangulat*) AND ((student* or pre-registration*) NEAR/4 (nurs*))

**Search strategy ProQuest global dissertations**

(suicid* OR parasuicid* OR para-suicid* OR overdos* OR nssi OR dsh OR selfinflict* OR selfharm* OR sdv OR selfmutilat* OR selfpoison* OR selfinjur* OR automutilat* OR hanging OR ligatur* OR strangulat*) AND ((student* or pre-registration*) NEAR/4 (nurs*))
